# Mechanical Thrombectomy for Stroke and COVID-19 Pandemic: A Critical Insight for Anesthesiologists

**DOI:** 10.3389/fmed.2021.674034

**Published:** 2021-07-22

**Authors:** Wesley Rajaleelan, Lashmi Venkatraghavan, Bernhard Schaller, Tumul Chowdhury

**Affiliations:** ^1^Department of Anesthesia and Pain Management, Sunnybrook Health Sciences Centre, University of Toronto, Toronto, ON, Canada; ^2^Department of Anesthesia and Pain Management, Toronto Western Hospital, University Health Network, University of Toronto, Toronto, ON, Canada; ^3^Department of Pathology, Institute of Cardiovascular Physiopathology, University of Buenos Aires, Buenos Aires, Argentina

**Keywords:** COVID-19, stroke, anesthesia, mechanical thrombectomy, safety

## Introduction

There is increasing evidence of a higher incidence of stroke in patients with coronavirus disease 2019 (COVID-19) infection (1, 2). This poses significant implications for anesthesiologists in the management of this complex patient population for emergency management of acute ischemic stroke (AIS). In this article, we would like to shed light on this topic by critically appraising the current literature specifically addressing anesthetic management during interventional treatment of ischemic stroke in patients with COVID-19.

### COVID and Stroke

During the start of the pandemic, a brief report claimed a decreasing trend of mechanical thrombectomy in Shanghai by almost 50% (3). This trend was surprising as it was expected that AIS will be reported as a possible complication of COVID-19 (4, 5). Cohorts from three hospitals in China showed that up to 36% of patients with COVID-19 infection had a variety of neurological symptoms including headache, dizziness, encephalopathy, and anosmia (3–5). Similarly, during the initial pandemic phase in Italy, a study by Lodigiani et al. (388 consecutive patients with COVID-19) pointed out that the thromboembolic complications following COVID-19 represented an integral part of the clinical picture of the neurological manifestations of this viral infection; however, the exact incidence might have been still underreported due to the low number of specific imaging tests performed (6). Strikingly, another study (pooled analysis of four studies) also highlighted the similar notion that there was a higher chance (up to 2.5 times) of severe COVID-19 illness in patients with symptomatic cerebrovascular disease (7).

The incidence of overall stroke during the pandemic was reported (mainly retrospective data) to be between 2.5% and 6% from China and Europe, and it is more likely to occur within the first 14 days following the COVID-19 diagnosis (2, 3). During the COVID outbreak in Wuhan, the study by Huang et al. (221 patients with COVID-19) reported that 5% of the patients presented with AIS, 0.5% developed cerebral venous sinus thrombosis, and 0.5% with cerebral hemorrhage (8). This may be due to the raised serum concentrations of the inflammatory cytokines that caused endothelial damage and dysfunction, increasing the pro-coagulant activity of the blood, which essentially contributes to the formation of a thrombus over a damaged arterial plaque (8). In addition, they also noted that COVID-19 patients with new onset of stroke were significantly older (71.6 ± 15.7 vs. 52.1 ± 15.3 years, *p* < 0·05) as compared to those not infected (8). Similarly, another study from Wuhan, China, reported 14 cases of stroke out of 219 patients with COVID-19 symptoms and further concluded that COVID-19 should be included in the differential diagnosis for patients with symptomatic cerebrovascular diseases (9). Looking at the various associations, a recent study of 46,248 patients with COVID-19 by Yang et al. revealed the two most prevalent comorbidities: hypertension (17%) and diabetes (8%). Both are also risks factors for stroke. Interestingly, cardiovascular disease accounted for only 5% of the patients, supporting an association between COVID-19 and stroke in a population without the typical vascular risk factors (10). They further concluded that the COVID-19-induced hypercoagulability was probably the most important mechanism of thrombosis in patients presenting with cerebrovascular symptoms. Thus, the higher incidence of cerebrovascular events was more likely due to the pronouncement of the underlying stroke-related characteristics than a new finding in COVID-19 patients (10).

The recently concluded STAR and the ENG trials in COVID-19 patients from 28 stroke centers in five countries reported that the median age distribution in patients presenting with stroke was 58 years, and there were no significant differences in the distribution with either gender or race. They also reported a low number of confirmed COVID-19 infections among patients with AIS undergoing mechanical thrombectomy. They concluded that intubation prior to mechanical thrombectomy during the early stages of stroke was associated with a greater in-hospital mortality and lower functional independence at discharge (11).

### Anesthesia and Mechanical Thrombectomy

Endovascular revascularization treatment remains the standard of care for AIS caused by large (cerebral) vessel occlusion in patients presenting within 6 h from the onset of symptoms of stroke. This is true even during the pandemic if patients meet specific neuroradiological criteria. There seems, however, to be an ongoing debate with regard to the ideal anesthetic technique for this procedure (1, 2). General anesthesia (GA) and conscious sedation (CS) have been described for patients undergoing endovascular thrombectomy (EVT) (Table 1). The advantages of GA included airway protection with lesser risks of pulmonary aspiration, patient immobility, and higher patient compliance. In contrast, local anesthesia or CS, with the patient spontaneously breathing, is associated with shorter procedure time and lower hemodynamic instability. However, the pandemic situation poses additional anesthesia risks.

**Table 1:** Pros and cons of general anesthesia and monitored anesthesia care in COVID-19 patient undergoing mechanical thrombectomy for ischemic stroke.

**General anesthesia**	**Local anesthesia**
**Pros** 1. Avoidance of patient movement 2. Airway protection 3. Avoidance of aerosol contamination during the case 4. Proetction of team 5. Delayed neurological examination	**Pros** 1. Better haemodynamic profile 2. Less chances of postoperative nause and vomiting 3. Access to neurological evaluation
**Cons** 1. Highere chances of hypotenisve episodes 2. Higher chances of postoperative nausea and vomiting 3. Potential for the time delay for starting the procedure 4. Risks of extubation induced aerosol contaimination 5. Increase wait time after the extubation (if in neuroangio suite)	**Cons** 1. Risk of airway compromise 2. Risks of image distortation and procedure failure (patient's movement related) 3. Risks of aerosol contamination if converted to GA or during coughing 4. Risks to team

Pooled data from multiple studies have shown that patients who underwent endovascular treatment under GA have worse outcomes compared to those with CS (12). Wan et al., in a recent meta-analysis of 6,703 patients, reported that patients in the GA arm had lower odds ratios (ORs) of favorable outcome when compared to those in the CS group (OR = 0.62, 95% CI = 0.49–0.77). Moreover, patients in the GA group were associated with a statistically significant higher risk of mortality (OR = 1.68, 95% CI = 1.49–1.90) (12). Brinjikji et al., in a meta-analysis, have suggested that the time delay associated with intubation could have led to worse outcomes for patients in the GA group (13). This meta-analysis, however, failed to prove non-significant or non-clinically relevant differences in most of the prespecified time intervals and procedure durations. Albeit the time intervals were shorter in the CS group, there were no significant differences in the groin puncture to reperfusion time or any differences in the total duration of the intervention found. Interestingly, they found that the mean time delay caused by the induction of GA compared to monitored anesthesia care (MAC) was only 6 min (13).

In contrast, a recent systematic review and meta-analysis of pooled data from four randomized control trials (RCTs) showed that patients who underwent EVT under GA had higher rates of successful recanalization and good functional outcomes at 3 months compared to patients treated with CS (14). The GA group also had non-significant trends toward a lower 3-month mortality. The proportions of patients with good functional outcomes at 3 months were 49.3% in the GA group and 36.6% in the CS group, an absolute difference of 12.7% (14). The value of these findings is not clear, as, in general, observational studies and meta-analyses have reported worse outcomes after GA when compared to those patients who have had CS. One explanation could be a selection bias in other studies compared to that in Campbell et al. (14). In summary, both GA and CS have been shown to be safe with good functional outcomes after mechanical thrombectomy. However, the choice of anesthetic technique still depends on the individual patient's condition and the institutional practice.

## Discussion

**Figure 1:**
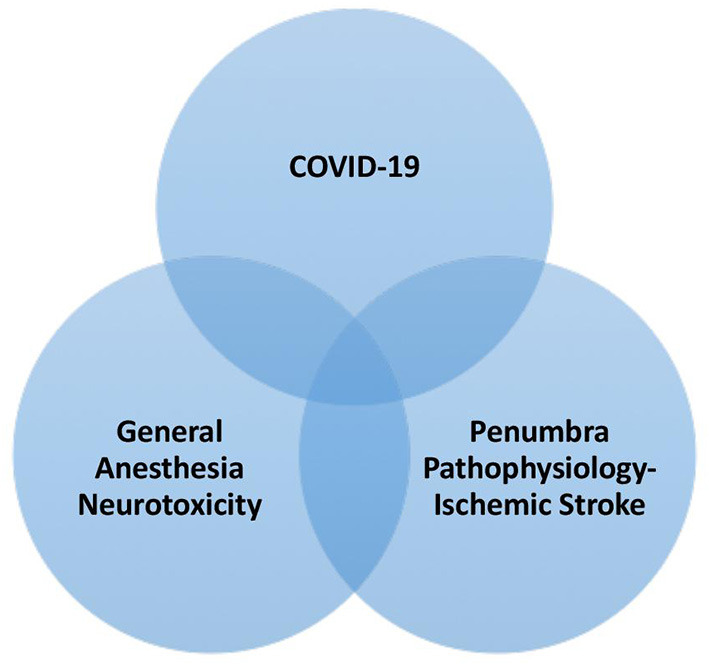
The influence of COVID 19 on ischemic stroke during general anesthesia.

There are minimal data on the outcome differences in patients with COVID-19. One of the main challenges with EVT during the pandemic was the risk associated with aerosol-generating medical procedures, such as airway management. Though avoiding GA may seem to be the choice in minimizing the risk of exposure, emergency airway management as a result of periprocedural complications increases the risk of exposure. The pathophysiology of stroke during anesthesia is not yet fully understood. However, they are cellular and molecular factors mediating GA-induced neurotoxicity and might be more prone during COVID-19 infection linked to reactive oxygen species (ROS) formation, mitochondrial permeability transition pore (mPTP) activation, increased Ca^2+^ influence, and increased tumor necrosis factor (TNF) and IL-1beta production (Figure 1).

Due to the increased chances of infectivity, and in order to minimize exposure and reduce the delay, it is recommended that patients presenting with a stroke during a pandemic should be directly referred to a tertiary care facility where EVT can be performed under MAC or GA in a negative pressure suite with ample availability of personnel protective equipment (PPE) kits without delay. Imaging and patient transport time should be kept to a bare minimum (14, 15).

Whether or not chest CT should be performed along with head CT in COVID-infected patients remains questionable. Li et al., in a recent study, revealed that 49 out of 51 COVID-infected patient's revealed COVID-19 findings on chest CT. The hallmark features were reported as ground glass opacities and consolidation with or without vascular enlargement, interlobular septal thickening, and air bronchogram signs. The chance of a missed diagnosis of COVID-19 in this study was found to be very low (3.9%) (16). The other argument can be made that, in such a subgroup of patients, performing chest CT for grading the severity of lung involvement can be helpful in the decision-making for choosing the type of anesthesia (GA *vs*. CS) for such procedures (16).

Hypoxia, which is common in patients with stroke, may have significant adverse effects on an already ischemic brain, especially after stroke. An ischemic brain does not compensate in cerebral circulation especially during hypoxia like a normal healthy brain does (17). The role of oxygen therapy in ischemic stroke remains controversial because of the failure of clinical trials to demonstrate its efficacy due to the oxygen-induced free radical injury. The role of therapeutic oxygen in stroke remains uncertain due to the lack of evidence regarding its benefits. Roffe et al., in a large single-blind randomized clinical trial of 8,003 adults from 136 participating centers in the United Kingdom, concluded that, among non-hypoxic patients with acute stroke, the prophylactic use of low-dose oxygen supplementation did not reduce death or disability at 3 months (17).

With regard to oxygen saturation in COVID-19 patients, Shenoy et al., in a recent meta-analysis, concluded that the revaluation of target oxygen saturation in COVID-19 patients is essential, both in the inpatient and outpatient settings. While conducting randomized control trials in the inpatient settings, a target SpO_2_ >96% (upper target PaO_2_ limit of 105 mm) *vs*. a target SpO_2_ of 92–95% would be complex in terms of logistics. In reality, an SpO_2_ in the upper end of 92–96% in both inpatients and outpatients with COVID-19 would be ideal (18).

Patients with signs and symptoms or with known exposure to COVID-19 should be meticulously assessed by an experienced airway specialist. The decision to intubate for an EVT must be justified to a patient's need for airway protection, the risk of exposure to the airway provider and the risk to other care providers, and the potential success of the EVT. If the patient requires an advanced airway post-EVT before leaving the interventional radiology (IR) suite, the endotracheal tube (ET) should be clamped before transferring onto an exhaust filtered transport ventilator or manual ventilation with two viral filters. Once admitted in the intensive care unit (ICU), as deemed essential, the patient should be extubated in a negative pressure environment with the airway providers sporting adequate PPE (19).

In a recent cross-sectional survey, Chowdhury et al. sent a questionnaire to 259 tertiary care stroke centers with neurointerventional facilities worldwide. They found that the number of stroke patients and EVT cases were reported to have decreased during the pandemic (19). Most participants reported conducting COVID-19 testing before (49%) or after (31%) the procedure; surprisingly, 20% of the centers did not test at all. Only 16% of the participating centers reported using a negative pressure room for the EVT (18). Strikingly, 50% of the participating centers reported no changes in the anesthetic management of AIS patients undergoing EVT during the pandemic (19). Most centers (71%) apparently reported that intubation of patients requiring GA for EVT during the pandemic was performed in the neurointerventional suite, followed by the emergency room (12%), a dedicated induction room outside the neurointerventional suite (11%), or in the ICU (6%) (20).

There are no current studies comparing the efficacy of GA vs. CS for mechanical thrombectomy in patients with diagnosed COVID-19. However, Sharma et al. published a consensus statement on behalf of the Society for Neuroscience in Anesthesiology and Critical Care (SNACC), and they recommended that, irrespective of the choice of anesthetic technique, airborne precautions have to be cautiously followed for all patients (16). Diagnostic testing to rule out COVID-19 should be carried out, when deemed feasible, without a delay in EVT (16). The use of PPE, which includes N95 masks and powered air-purifying respirators (PAPRS), would be mandatory when performing airway manipulative procedures in patients with a known or suspected COVID-19 (16). They also recommended that the choice of anesthetic technique for EVT should be cautiously individualized for each patient, taking the patient's overall neurological and general status into account (16). In centers practicing CS for EVT, the threshold for the use of GA for EVT may be reduced during an active COVID-19 pandemic. Having stated this, not all patients presenting with a stroke would warrant a GA, as GA is associated with a risk of aerosol production. Airway interventions like intubation would require additional time taken to don and doff the PPE, and that might account for the delay in skin puncture time and revascularization (16). Sharma et al. also recommended that the most experienced anesthesiologist in the team should manage the airway. A closed-loop communication between the anesthesiologist and the interventional neuroradiologists with regard to the use of GA vs. MAC is of utmost importance. If the patient warrants a GA, then its induction should be carried out in an airborne isolation room equipped with negative pressure suites. The decision to proceed with induction and GA should be made early to avoid delays in puncture time and revascularization (15).

Smith et al. suggested that the decision to intubate a patient for EVT must be a delicate balance that would justify the patient's need for a definitive airway, the risks involved for the personnel, the ventilator capacity of the hospital system, and the success of the procedure, which would establish cerebral perfusion (19). They also recommended that the intubation should be carried out in a negative pressure induction room if the interventional radiology suite is not equipped with negative pressure air systems, backed by institutional protocols and the Centers for Disease Control and Prevention (CDC) guidelines (19). Once a definitive airway is established, they also recommended that the patient is transported to the IR suite with transport ventilators equipped with exhaust port filters, being cautious of circuit leaks and disconnections (19).

## Conclusion

All in all, data supporting an association between COVID-19 and stroke in populations without typical vascular risk factors are increasing. It seems that these patients are older and COVID-19 might not influence stroke solely through a single mechanism that might have implications well-beyond the clinical condition of stroke or related interventions. For managing such patients, there are three critical points to be considered. Firstly, anesthetic management in such patients should be individualized. Secondly, the anesthetic technique that is standard practice at the institution should still be the first choice. Finally, for the safety of the team, proper simulation, standard donning and doffing of PPE, and effective communication should be employed. Ideally, larger prospective studies are necessary to discuss the anesthetic management challenges in these patients. An awareness and knowledge of the underlying factors of these issues are paramount for the entire stroke team, including anesthesiologists, caring for this growing patient population.

## Author Contributions

WR has contributed significantly in writing and preparing this manuscript. LV and BS have contributed in providing critical insight and writing and editing the manuscript. BS has also contributed in making table and figure. TC is the corresponding author and contributed significantly in designing, conceptualizing, writing, and editing and providing critical insight. All authors contributed to the article and approved the submitted version.

## Conflict of Interest

TC serves as an Associate Editor for Frontiers in Medicine. TC and BS both have served as Guest Associate Editor for Autonomic Neuroscience, Frontiers in Neuroscience, Frontiers in Neurology, and Frontiers in Physiology. The remaining authors declare that the research was conducted in the absence of any commercial or financial relationships that could be construed as a potential conflict of interest.

## Abbreviations

GA: general anesthesia
COVID 19: corona virus disease 2019
CS: conscious sedation
OR: odds ratio
CI: confidence interval
AIS: acute ischemic stroke
RCT: randomized control trial
EVT: endovascular thrombectomy
PPE: personnel protective equipment
PAPRS: powered air purifier respirators
N95—: This is a Respirator Rating Letter Class.It stands for “Non-Oil” meaning that no oil-based particulates are present.
